# Radiofrequency Catheter Ablation for Atrial Fibrillation: Low-power, Long-duration Versus High-power, Short-duration

**DOI:** 10.19102/icrm.2024.15086

**Published:** 2024-08-15

**Authors:** Luka Petrovic, Bharat K. Kantharia

**Affiliations:** 1 Section of Cardiac Electrophysiology, Mount Sinai Hospital—Morningside, St. Lukes, New York, NY, USA; 2 Icahn School of Medicine at Mount Sinai, New York, NY, USA; 3 Cardiovascular and Heart Rhythm Consultants, New York, NY, USA

**Keywords:** Ablation power, atrial fibrillation, catheter ablation, umbrella review.

Catheter ablation of atrial fibrillation (AF) is an effective rhythm-control strategy that achieves many beneficial outcomes, including reductions in mortality, heart failure, and health care usage.^1–3^

Fundamentally, to achieve a successful outcome of AF ablation with durable electrical isolation of the pulmonary veins (PVs) and other arrhythmogenic foci in the atria, it is imperative that the ablation lesions have transmurality and are linearly contiguous, without any electrically active gaps between the lesions. When radiofrequency (RF) thermal energy is selected, good-quality ablation lesions are dependent on RF power and current delivery and total duration of the energy application as well as surface area of the catheter tip; adequate stability, contact, and orientation of the catheter; and mechanisms such as saline irrigation for dissipation and deeper penetration of thermal energy.^4,5^ Furthermore, ablation lesions must also not lead to complications, such as steam pops, damage to collateral cardiac and non-cardiac structures, perforation, or PV stenosis.^6^ To accomplish such goals, RF energy is applied either at a low power for a long duration (LPLD) or at a high power for a short duration (HPSD). There is, however, no uniform or single ablation strategy, and the decision about whether to use LPLD or HPSD falls under the operator’s discretion. There is also no consensus about a clear definition in terms of exact power cutoffs, although LPLD and HPSD have been characterized generally to involve a power of 20–40 W for a relatively long duration (20–40 s) and a power of ≥50 W for a short duration (5–10 s), respectively.^7–10^ Thermal injury during RF ablation occurs during resistive heating and conductive heating. Resistive heating represents injury that happens due to immediate heating of the tissue layer in contact with the catheter, while conductive heating is the consequence of the passive extension of heat created by resistive heating into the deeper tissue layers. Irreversible cellular death occurs at temperatures that are ≥50°C, while reversible injury occurs at a lower temperature.^9^ The HPSD approach allows the uniformity of transmural lesions by increasing the effect of resistive heating that would produce direct uniform injury to the tissue. Furthermore, by minimizing the conductive heating, HPSD is considered to limit damage to the collateral structures and minimize the complications related to RF ablation.^9,10^ Reconnection of the PVs after successful PV isolation (PVI) is considered to occur due to the partial thickness of lesions and reversible injury.^11,12^

In the current issue of *The Journal of Innovations in Cardiac Rhythm Management*, Pavani et al.^13^ report an umbrella review of existing systematic reviews and meta-analyses to assess the safety and efficacy of HPSD ablation. Pertaining to the methodology, after identifying 35 systematic reviews and meta-analyses, the authors narrowed them down to a final selection of 11 studies that collectively integrated data from 6 randomized controlled trials and 26 observational studies for an umbrella review. The investigated clinical outcomes were atrial tachyarrhythmias and AF recurrence after the blanking period of 2–3 months, esophageal thermal injury, and other major complications. Secondary outcomes that were included in the study were first-pass PVI, acute PV reconnection, PVI number, procedural time, and fluoroscopy time. The definition of high power for the purpose of the umbrella review was a power of >40 W. Atrial arrhythmia recurrence was defined as episodes of post-ablation atrial arrhythmia lasting >30 s after the blanking period. Esophageal thermal injury was evidenced through endoscopy and/or magnetic resonance imaging. The main findings of the study include (1) no significant difference in the recurrence of atrial tachyarrhythmias between HPSD and LPLD strategies (risk ratio [RR], 0.88; 95% confidence interval [CI], 0.70–1.12; *P* = .31), (2) significantly less AF recurrence with the HPSD approach (RR, 0.53; 95% CI, 0.42–0.67; *P* < .00001), (3) less esophageal thermal injury with the HPSD approach versus the LPLD approach (RR, 0.71; 95% CI, 0.61–0.83; *P* < .00001), and (4) no significant difference in other major complications between the groups (RR, 0.87; 95% CI, 0.73–1.03; *P* = .10), respectively.^13^ Furthermore, besides a higher first-pass PVI rate, other secondary outcomes, ie, total procedural time, fluoroscopy time, and ablation time, were also better with the HPSD approach compared to the LPLD approach.^13^

The authors are to be commended for exploring a highly discussed topic in the sphere of catheter ablation of AF. The findings of the study that show decreased AF recurrence after the blanking period and a decrease in the chance of an esophageal thermal injury support the concept that HPSD catheter ablation does increase the durability of the RF lesions and also limits the potential for collateral damage by modification of the ratio of resistive and conductive heating when compared to LPLD. The limitations of the study are related to the quality and heterogeneity of the primary data used in the included systematic reviews and meta-analyses and the lack of a uniform definition of high power. The latter point is in line with the shortcomings of an umbrella review, which, as a research method, employs a systematic collection of multiple systematic reviews or meta-analyses that are done on a particular topic of interest.^14^ While an umbrella review provides several advantages, such as saving resources by avoiding repetitious searches, there are certain disadvantages, including the fact that the validity of the findings depends on the quality of the included systematic reviews and meta-analyses.

The scientific application of the HPSD approach can be questioned for comparative efficacy and safety against those of some other thermal ablation approaches, including very-high-power ablation; cryoablation; and, of course, non-thermal pulsed-field ablation (PFA). Thermal RF ablation energy may now be delivered at a very high power of 90 W for a short amount of time (4 s) with the recently introduced QDOT MICRO™ catheter (Biosense Webster, Diamond Bar, CA, USA) with specialized thermocouples at the catheter tip. As reported in the FAST and FURIOUS studies, it is now possible to perform not only AF ablation but also ablation of other cardiac arrhythmias, including focal and macro–re-entry atrial tachycardia, typical cavotricuspid isthmus-dependent atrial flutter, premature ventricular contractions, and accessory pathways, effectively and safely with the QDOT MICRO™ catheter using a very-high-power, short-duration ablation approach.^15^ The contemporary role of RF energy is also of a dynamic nature given the introduction of PFA as the energy source. The novel, catheter-based, non-thermal PFA approach uses electroporation to increase the permeability of cell membranes and induce cardiac cell death via apoptosis.^16,17^ The efficacy and safety profile of PFA as reported through large studies have enabled regulatory approval in the United States and the European countries.^16,17^ Furthermore, with the added short procedural time and more efficient workflow, more centers are switching to PFA to treat not only patients with paroxysmal AF requiring PVI alone but also those with post-ablation recurrence of AF and/or persistent AF in whom the strategy of substrate modification, eg, posterior wall isolation, is required.

While more experience is being gained with the newer technologies and ablation energy sources, until they become freely available, there remains the necessity to continue using thermal ablation with RF energy. In that respect, we believe that the knowledge imparted by studies such as the one discussed here by Pavani et al.^13^ may help electrophysiologists in their quest for better outcomes of AF ablation.
